# Factors influencing motivation of nurse leaders in a private hospital group in Gauteng, South Africa: A quantitative study

**DOI:** 10.4102/curationis.v43i1.2011

**Published:** 2020-02-27

**Authors:** Maria Breed, Charlene Downing, Hafisa Ally

**Affiliations:** 1 Department of Nursing, University of Johannesburg, Johannesburg, South Africa

**Keywords:** motivation, leadership, unit managers, quantitative research, nurse leaders.

## Abstract

**Background:**

Nurse leadership is about aligning employees to a vision. This happens with buy-in, motivation and communication. When conducive environments are created by organisations, the motivation of nurse leaders will be enhanced, which will have a positive outcome on the organisation. Highly motivated nurse leaders accomplish more and are more productive. Nurse leadership is an essential source of support, mentorship and role modelling. These attributes tend to be more evident when nurse leaders are motivated.

**Objectives:**

The objective of this study was to determine the factors that influence the motivation of nurse leaders.

**Method:**

A quantitative, descriptive design and stratified sampling was used. Participants comprised unit managers (*n* = 49) from five hospitals in a private hospital group in South Africa. A self-administered questionnaire, namely, the Multidimensional Work Motivation Scale, was used to collect the data. Data were analysed using the IBM SPSS 22.0 program.

**Results:**

The results indicated that the nurse leaders in this study were intrinsically motivated. Their motivation was influenced by support, relatedness, autonomy and competence. No relationships were found between motivation and age, years in a management position, gender, qualifications and staff-reporting structure.

**Conclusion:**

By implication, to understand what motivates nurse leaders and to keep them motivated, recommendations were proposed to nursing and human resources management. It is expected that the implementation of the recommendations will have a positive influence on patient outcomes, organisational success and the motivation and satisfaction of nurse leaders.

## Introduction

Leadership is a concept of importance on a global level, as the success of any organisation depends on the quality of its leadership. Today’s nurse leaders must motivate and engage a diverse and multigenerational labour force, ranging from employees on their payroll to part-time workers employed on a contract basis (Jesuthasan & Holmstrom 2017). According to Zhang, Fan and Zhang (2015), leaders with a high degree of motivation take the initiative, seek responsibilities, have a positive attitude regarding taking risks and are eager to learn.

Leadership can be described as the ability to organise and influence employees with specific skills to complete delegated tasks to achieve results (Ramchunder & Martins 2014). Leaders formulate a common vision, motivate others and offer stability during times of transformation (Martin 2015). According to Cooper (2015), leadership is the ability to influence others to achieve organisational goals. Ramchunder and Martins (2014) believed that the leader’s ability to influence the behaviour of employees can positively influence performance outcomes.

This study concerns the concept of motivation as influenced by leadership, specifically leadership of unit managers. Unit managers are the first-level leaders in nursing. Motivation is the leader’s degree of preparedness to apply and preserve an effort to achieve the organisation’s goals (Akintola & Chikoko 2016). Motivation is a critical part of leadership as people need to understand each other to be effective leaders. This motivation is a process that directs and influences behaviour (Jooste & Hamani 2017). Motivation is essential in providing nurse leaders with reasons for a certain behaviour and plays an important role in guiding behaviour and decision-making (Frielink, Schuengel & Embregts 2017).

Nurse leaders display different types of motivation. These are driven by three basic needs: autonomy, relatedness and competence (Allan et al. 2016). The need for autonomy and competence relates to engaging with tasks that leaders find interesting and promote growth in their autonomy and competence (Shu 2015). Relatedness is the extent to which nurse leaders feel they are cared for and connected to others. Consequently, relatedness creates feelings of belonging and provides a sense of safety. Nurse leader’s competence, on the contrary, refers to a leader’s gifts and abilities in a specific domain (Conway et al. 2015).

Because of the high demands that are placed on nurse leaders to produce outcomes and remain professional and motivated, it is important to identify what motivates nurse leaders and provide recommendations to enhance their motivation or keep them motivated. Studies have been conducted on motivation (Akintola & Chikoko 2016) and leadership (Kantanen et al. 2017) in general, but the first author has found a gap in specific research on nurse leaders, specifically concerning nursing unit managers and their motivation in South Africa. No evidence of specific research on nursing leadership, specifically regarding the motivation of unit managers in the private hospital sector, has been found.

### Theoretical framework

This study is based on the Self-Determination Theory. This theory assists in understanding employees’ motivation in the work setting, and focusses on the degree to which needs are satisfied and not necessarily individual differences in needs’ strength as discussed by Graves and Luciano (2013:520). It also differentiates between different types of motivation, which falls along a continuum of self-determination from intrinsic, integrated, identified, introjected, extrinsic and amotivation (Jochems et al. 2014:495).

Intrinsically motivated work behaviour creates congruence between work behaviours and one’s self-concept, which results in feelings of meaningfulness (Allan, Autin & Duffy 2016). This type of motivation is regulated by personal enjoyment, interest or pleasure, and it involves the performance of an activity for the inherent satisfaction of the activity (Naile & Salesho 2014:177). Putting effort into a job is interesting and exciting.

Introjected motivation drives action to avoid guilt and shame and enhance the ego (Battistelli et al. 2015). Introjected motivation is somewhat less controlled and represented by behaviours driven by internal rather than external rewards. The leader is motivated to avoid self-conscious emotions and obtain positive self-regulated affects and appraisals (Nie et al. 2015:246). Putting effort into a job in order not to feel bad or ashamed about one’s self, even taking the risk of losing one’s job if it is not satisfactory.

In identified motivation, actions are performed because such actions are personally important and valuable to the nurse leader (Maulana et al. 2016). Putting effort into one’s job because you consider it important, because the job aligns with personal values, and because it has personal significance.

Extrinsic motivation arises from the influence of external activities that direct nurse leaders to perform to get rewards in return (Hee & Kamaludin 2016), and amotivation refers to the total lack of any intention to act (Nie et al. 2015). Putting effort into one’s job to get other people’s approval, other people’s respect and to avoid criticism are examples of this motivation.

Motivation tends to differ in leaders who are engaged versus disengaged; therefore, the need to elaborate on these concepts. Engaged leaders motivate behaviour by changing basic values, beliefs, attitudes and assumptions of employees by raising their awareness of organisational goals (Wipulanusat, Panuwatwanich & Stewart 2017). It can be argued that disengaged leaders are less motivated and therefore not able to motivate their employees (Jooste & Ntamane 2014).

The factors that influence the motivation of nurse leaders must be identified to implement targeted strategies for continuous improvement (Zarei et al. 2016: 2250). The purpose of this study was to fill this identified gap by determining the factors that influence nurse leader’s motivation. There appears to be no literature that specifically deals with this topic in terms of nurse leaders and therefore the need to address this gap.

## Aim and objectives

The purpose of this study was to determine factors that influence the motivation of nurse leaders.

## Research method and design

### Research design

A quantitative research design was applied. A quantitative design is defined by Fouché et al. (2014: 64) as an inquiry into a social or human problem, based on testing a theory consisting of variables, measured with numbers and analysed with statistics. The first author conducted an investigation into the factors that influence the motivation of nurse leaders – variables were identified and statistics were analysed, thus leading to the decision to use a quantitative research design. In this study, the opinions of the nurse leaders were taken as a representation of the truth, as their opinions reflected their perceptions of the construct within this study. This research design was effective in obtaining knowledge about nurse leaders’ motivation as very little literature is available about the behaviour with specific reference to nurse leaders.

### Setting

This study was conducted at five different hospitals of one private hospital group in the Gauteng province in South Africa.

### Study population and sampling strategy

The respondents included nurse leaders identified as unit managers. Stratified sampling was used, and all the unit managers from the selected hospitals in the northern region of Gauteng in South Africa had an equal opportunity to participate. Each hospital had about 12 unit managers on their payroll, and they were included. The sample size amounted to 60 (*N* = 60). The criteria for the unit managers to be included in the study as the target population were as follows: unit managers (1) had to be in a managerial position for more than 6 months, (2) had to be responsible for more than five staff members and (3) had to be working in a private hospital setting of a specific group. Different hospitals were selected by means of the probability sampling method; a simple random sampling method was used. The total accessible population size was *N* = 60. A total of 49 questionnaires were completed and returned. The return rate was 82%.

### Data collection

A two-part questionnaire consisting of 40 questions using a Likert scale, with options ranging from *strongly disagree, disagree, undecided* and *agree to strongly agree*, was used. The Likert scale most commonly addresses response choices such as agreement, evaluation or frequency (Burns, Gray & Grove 2013). In this questionnaire, the response choice was for agreement. A self-administered questionnaire was used, using questions from the Multidimensional Work Motivation Scale, the Work-Related Basic Need Satisfaction Scale and the Perceived Organisational Support Survey, to collect the data.

All unit managers in the selected hospitals were invited to complete the self-administered questionnaire. Sixty questionnaires were distributed, either electronically or by hand. Section A included a cover letter explaining the purpose of the questionnaire, the first authors’ name and the supervisors involved, and the affiliated university.

Section B consisted of guidelines and explanations on how to complete the questionnaire. Section B consisted of two sections, and section 1 included biographical questions. Table 1 shows the components addressed in section 2 of the questionnaire.

**TABLE 1:** Components addressed in part 2 of the questionnaire.

No. of questions	Items included	Scale used	Authors
17	Autonomy, competence, relatedness	Work-Related Basic Need Satisfaction Scale	Van den Broeck et al. (2010)
4	Support	Perceived Organisational Support Survey	Rhoades, Eisenberger and Armeli (2001)
3	Amotivation	Multidimensional Work Motivation Scale	Gagné et al. (2014)
3	Extrinsic regulation – social	Multidimensional Work Motivation Scale	Gagné et al. (2014)
3	Extrinsic regulation – material	Multidimensional Work Motivation Scale	Gagné et al. (2014)
4	Introjected regulation	Multidimensional Work Motivation Scale	Gagné et al. (2014)
3	Identified regulation	Multidimensional Work Motivation Scale	Gagné et al. (2014)
3	Intrinsic motivation	Multidimensional Work Motivation Scale	Gagné et al. (2014)

Data collected in previous studies with this instrument showed the scales to be valid and reliable (Gagné et al. 2014; Rhoades et al. 2001; Van den Broeck et al. 2010). Cronbach’s alpha coefficient is the statistical procedure used for calculating internal consistency (Burns et al. 2013).

In the above-mentioned studies, the Cronbach alpha for autonomy, competence and relatedness was on average more than 0.80 (Van den Broeck et al. 2010). This indicates that the instrument is 80% reliable with 20% random error. For support, it was measured between 0.74 and 0.80 (Rhoades et al. 2001), which indicates that the scale has a 74% – 80% reliability for the questions regarding support, and for different types of motivation it was measured above 0.70, which shows a 70% reliability score (Gagné et al. 2014).

### Data analysis

Data were analysed statistically by means of the IBM SPSS 22.0 program. The findings of the study were presented both as descriptive and inferential statistics. The descriptive statistics were presented as frequencies (*f*) that refer to the number of responses (*n*) on items using a five-point Likert scale (*n* = 49); the mean (*x˙*) of each item that will be presented in a table format from the highest to the lowest mean value; and the standard deviation (SD) of each item. The following descriptive statistics were analysed: autonomy, competence, relatedness, support, amotivation, extrinsic regulation – social, extrinsic regulation – material, introjected regulation, identified regulation and intrinsic motivation as an aspect of motivation. Inferential statistics were derived at by means of factor analysis and statistical significance. The varimax principal component analysis was used for factor analysis. Factor analysis was conducted on the responses between the following aspects of nurse leaders and motivation: age of the respondents, years in a managerial position of the respondents, gender of the respondents, level of qualification of the respondents and staff-reporting structure to the respondents.

### Ethical considerations

The respondents were made aware that participation was not compulsory and that they could withdraw at any stage. Each respondent signed written consent that they were participating voluntarily. No compensation was offered to any respondent. Permission to use the questionnaire was obtained from the relevant parties. Ethical approval was obtained from the University of Johannesburg Higher Degrees Committee (HDC-01-168-2015) and the Research Ethics Committee (REC-01-243-2015).

## Results

Table 2 indicates a breakdown of the characteristics of the respondents and the aspects that were investigated. In this study, age, gender, highest qualification, years in a managerial position and staff-reporting structure did not have an impact on the respondents’ motivation.

**TABLE 2:** Aspects investigated as an influence on nurse leaders’ motivation.

Age group			Staff-reporting structure			Gender			Highest qualification			Years in a managerial position		
Age	*n*	%	Number of staff	*n*	%	Gender	*n*	%	Qualification	*n*	%	Year	*n*	%
31–40	10	20	1–5	1	2	Male	3	6	Diploma	32	65	< 1	6	12
41–50	23	47	6–10	4	8	Female	46	94	Degree	17	35	1–2	6	12
51–60	13	27	11–15	7	14	-	-	-	-	-	-	2–3	5	10
61+	3	6	16–20	12	25	-	-	-	-	-	-	3–4	3	6
-	-	-	> 20	25	51	-	-	-	-	-	-	4–5	2	4
-	-	-	-	-	-	-	-	-	-	-	-	5–6	7	15
-	-	-	-	-	-	-	-	-	-	-	-	6–7	1	2
-	-	-	-	-	-	-	-	-	-	-	-	7+	19	39

The ages of respondents were between 31 and 62 years. It was established from the data obtained that the largest group was between 41 and 50 years (47%). The smallest age group was the group older than 61 years (*n* = 3), which accounted for 6%. The group with ages between 31 and 40 years constituted 20% of the respondents, and the third group identified was the age group of 51–60 years, which totalled 27% of the respondents.

The highest qualified leaders were in the age group of 50–59 years who will soon enter retirement age and will leave a gap in the intellectual capital. The age group with the highest amount of degrees was between 50 and 59 years; this group had eight degrees (16%), while the group between 40 and 49 years had six degrees (12%) and the group between 31 and 39 years had three degrees (6%). The age group older than 60 years had no degrees. Only 17 of the 49 respondents, which is 35%, had a degree.

Only 6% of the respondents were men who participated; this illustrates that the nursing leadership roles are predominantly led by women. As shown in the Table 2, the majority of the respondents (39%), (*n* = 19), were in a managerial position for more than 7 years. The majority of the respondents (51%), (*n* = 25), had more than 20 staff members reporting to them. The demographic information is shown in Table 2.

The descriptive analysis revealed that the following aspects influenced nurse leaders’ motivation. Influencing factors consisted of five items (support, relatedness, competence 1, autonomy relatedness and competence 2), and five motivation factors (identified regulation, extrinsic regulation – social, amotivation, intrinsic motivation and introjected motivation) were shown to influence motivation in nurse leaders.

### Relatedness or sense of belonging

With a mean value of 4.4694, item 2(at work I feel part of the group) showed the highest mean value – 98% either strongly agreed or agreed that they feel part of the group. The results indicate that the majority of the respondents felt related at their job. Table 3 shows an illustration of the results.

**TABLE 3:** Descriptive analysis of factors influencing motivation.

Relatedness	1 Strongly disagree		2 Disagree		3 Undecided		4 Agree		5 Strongly agree	
	*n*	%	*n*	%	*n*	%	*n*	%	*n*	%
At work, I feel part of the group (item 2)	-	-	1	2	-	-	23	47	25	51
I do not really feel connected with other people at my job (item 1)	23	47	23	47	1	2	1	2	-	-
I do not really mix with other people at my job (item 3)	20	41	23	47	4	8	2	8	-	-
I often feel alone when I am with my colleagues (item 5)	19	39	25	51	2	4	2	4	1	2
At work, I can talk with people about things that really matter to me (item 4)	2	4	5	10	7	14	20	41	15	31
Some people I work with are close friends of mine (item 6)	6	12	13	27	7	14	17	35	6	12
**Competence**										
I feel competent in my job (item 8)	-	-	-	-	-		15	31	34	69
I do not really feel competent in my job (item 7)	33	67	15	31	-	-	-	-	-	-
I doubt whether I am able to execute my job properly (item 9)	31	65	14	29	1	2	2	4	-	-
I feel that I can be myself at my job (item 12)	-	-	1	2	1	2	23	47	24	49
I am good at the things I do in my job (item 10)	1	2	-	-	1	2	25	51	22	45
I have the feeling that I can accomplish the most difficult tasks at work (item 11)	1	2	1	2	2	4	24	49	21	43
**Autonomy**										
The tasks I have to do at work are in line with what I really want to do (item 15)	2	4	-	-	2	4	28	57	17	35
I feel free to do my job the way I think it could best be done (item 16)	1	2	5	10	5	10	19	39	19	39
In my job, I feel forced to do things I do not want to do (item 17)	14	29	19	39	8	16	6	12	2	4
At work, I often feel like I have to follow other people’s instructions (item 13)	8	16	16	33	10	20	13	27	2	4
If I could choose, I would do things differently at work (item 14)	2	4	16	33	3	6	22	45	6	12
**Support**										
Nursing management cares about my well-being (item 19)	1	2	4	8	5	10	25	51	14	29
Nursing management shows very little concern for me (item 21)	11	22	27	55	4	8	7	15	-	-
Nursing management strongly considers my goals and values (item 20)	-	-	7	14	6	12	24	49	12	25
Nursing management cares about my opinions (item 18)	3	6	3	6	6	12	27	55	10	20

### Competence

This refers to feeling a sense of capability in the leader’s own ability to relate with their environment as well as obtaining opportunities to express capacities on a regular basis (Allan et al. 2016). Six items were included.

Item 8, which read ‘I feel competent in my job’, scored the highest mean value (*x˙* = 4.6939). All the 49 respondents either strongly agreed or agreed with this statement. In other words, most of the respondents felt that they were competent in their job and were certain about their capabilities and competencies. Table 3 shows the results as discussed.

### Autonomy

Autonomous motivation is a form of self-regulation whereby leaders act as a result of their deep values, goals and interests (Graves & Luciano 2013). Five items were included.

The highest mean value (*x˙* = 4.1837) was scored for item 15 (The tasks I have to do at work are in line with what I really want to do), where 92% either strongly agreed or agreed that they were doing tasks at work which were in line with what they wanted to do.

Most of the responses were positive in that the nurse leaders did feel a sense of autonomy. Table 3 shows the results for this section.

### Support

Strong and supportive leadership is a strong predictor of leader’s motivation and morale (Chipeta et al. 2016). Support consisted of four items.

Item 19, ‘Nursing management cares about my well-being’, scored the highest mean value (*x˙* = 3.96). Of the respondents, 10% strongly disagreed or disagreed, 10% were undecided, while 80% either agreed or strongly agreed. This means that the majority (80%) felt that this statement was true, and that 20% felt that management did not care about their well-being.

It can thus be concluded that the majority of the respondents felt that the management supported them. Table 3 shows the results for this section as discussed.

### Motivating factors

#### Amotivation

Amotivated people usually feel disengaged and helpless in doing activities and will therefore easily quit an activity or task (Chen & Bozeman 2013). A leader lacking motivation will thus only have a minimum level of determination to work (Rizal et al. 2014). Three statements were included.

The highest mean value (*x˙* = 1.31) was scored for item 22 (I will not put effort into my job because I really feel that I am wasting my time at work). All 49 respondents either strongly agreed or agreed with these statements, that is, they all agreed that they were not wasting their time at work and therefore would make an effort at work. After analysing these data, it can be said that not one of the respondents was a motivated. Table 4 shows the results for this section.

**TABLE 4:** Motivation factors influencing nurse leaders.

Amotivation	1 Strongly disagree		2 Disagree		3 Undecided		4 Agree		5 Strongly agree	
	*n*	%	*n*	%	*n*	%	*n*	%	*n*	%
I will not put effort into my job because I really feel that I am wasting my time at work (item 22)	34	69	15	31	-	-	-	-	-	-
I do little because I do not think it is worth putting effort into (item 23)	36	74	13	26	-	-	-	-	-	-
I do not know why I am doing this job, it is pointless work (item 24)	39	80	9	18	1	2	-	-	-	-
**Extrinsic regulation – social**										
I put effort into my job because other people will respect me more (item 26)	5	10	20	42	4	8	9	19	10	21
I put effort into my job to get other people’s approval (item 25)	7	15	21	43	2	4	10	20	9	18
I put effort into my job to avoid being criticised by others (item 27)	12	25	19	39	3	6	7	14	8	16
**Introjected regulation**										
I put effort into my job because it makes me feel proud of myself (item 32)	-	-	1	2	1	2	5	10	42	86
I put effort into my job because I have to prove to myself that I can do it (item 31)	3	6	6	12	2	4	8	16	30	62
I put effort into my job because otherwise I will feel bad about myself (item 34)	1	2	8	6	2	4	18	37	20	41
I put effort into my job because otherwise I will feel ashamed of myself (item 33)	1	2	10	20	3	6	15	31	20	41
**Identified regulation**										
I put effort into my job because putting effort into this job aligns with my personal values (item 36)	-	-	-	-	-	-	7	14	42	86
I put effort into my job because I personally consider it important to put effort into this job (item 35)	-	-	-	-	-	-	10	20	39	80
I put effort into my job because putting effort into this job has personal significance for me (item 37)	-	-	-	-	-	-	11	22	38	78
**Intrinsic motivation**										
I put effort into my job because the work I do is interesting (item 40)	1	2	1	2	4	8	12	5	30	63
I put effort into my job because I have fun doing my job (item 38)	1	2	-	-	2	4	22	45	24	49
I put effort into my job because what I do in my work is exciting (item 39)	1	2	1	2	2	4	23	47	22	45

#### Extrinsic regulation – social

Extrinsic motivation is an external force, leading the nurse leader to meet personal and organisational goals. This occurs because of external activities, such as pressure or instruction, which influence leaders to perform tasks and reap the rewards in return (Hee & Kamaludin 2016). Here three items were included.

Item 26, ‘I put effort into my job because other people will respect me more’, scored the highest mean value (*x˙* = 2.98): 40% strongly agreed or agreed with this statement, 8% were undecided and 52% either disagreed or strongly disagreed. A majority of respondents (52%) disagreed with this statement, which indicates that gaining the respect from others was not a motivating factor for the respondents.

This analysis indicates that extrinsic motivation, especially the social components of motivation, did not play an important role for the respondents (see Table 4 for an illustration of these results).

#### Introjected regulation

This type of motivation refers to motivation arising from a desire to satisfy the demands from others, that is, acting to avoid feelings of guilt or out of a psychological need to prove something (Gaston et al. 2016). Introjected regulation consisted of four items.

Item 32, ‘I put effort into my job because it makes me feel proud of myself’, scored the highest mean value (*x˙* = 4.80). This statement regarded pride in oneself because of a job done. Of the 49 respondents who responded, 96% strongly agreed or agreed with the statement, 2% were undecided and 2% disagreed. After analysing the data, it could be established that introjected regulation was an important motivator for the respondents. Table 4 shows an indication of these results.

#### Identified regulation

Identified regulation represents the lower end of the spectrum regarding autonomous motives and refers to motivation arising from a longing to accomplish a result, which is personally valued by the leader (Gaston et al. 2016). This consisted of three items.

Item 36, ‘I put effort into my job because putting effort into this job aligns with my personal values’, had the highest mean value (*x˙* = 4.86). Of the 49 respondents, 86% strongly agreed and 14% agreed. This showed that personal values and job alignment were important aspects for the respondents. The analysed data thus showed that identified regulation is an important factor of motivation. Table 4 shows the results of this section.

#### Intrinsic motivation

Intrinsic motivation is an internal force that leads leaders to meet personal and organisational goals. This type of motivation can be described as inherently interesting and enjoyable that creates behaviour and encouragement to act (Hee & Kamaludin 2016). Three items were included.

Item 40, ‘I put effort into my job because the work I do is interesting’, scored the highest mean value (*x˙* = 4.44). A majority of 68% agreed with this by either strongly agreeing or agreeing, 8% were undecided and 4% disagreed with this. Item 38 was about having fun while doing the job: 94% agreed with this, 4% were undecided and the remaining 2% strongly disagreed.

Thus, most of the responses showed that the respondents favoured intrinsic motivation and regarded it as an important component of their motivation level. In Table 4, the results for this section are presented.

For this study, the Cronbach alpha for autonomy was 0.660; for competence, it was 0.770; and for relatedness, it was 0.732. For support, it was 0.886. The different types of motivation scored as follows: amotivation, 0.828; extrinsic regulation – social, 0.847; extrinsic regulation – material, 0.793; introjected regulation, 0.523; identified regulation, 0.878; and intrinsic motivation, 0.920.

## Discussion

Age, years as a manager, qualification and number of staff reporting to the nurse leaders do not seem to be potential predictors of autonomy, competence and relatedness, and do not serve as motivation factors in this study on nurse leaders. Support was identified as one of the important aspects of motivation for nurse leaders, as they needed to feel that they were cared for and that their well-being was important to the organisation.

Relatedness was identified as another aspect that influenced motivation in nurse leaders, and therefore it was important for them to feel part of the team and that they could connect with others at work. In this way, they did not feel isolated and alone. It is thus important that nurse leaders feel that they have someone to talk to, as this will enable them to voice their opinions and be autonomously motivated. Competence is an important aspect of motivation, and leaders need to be empowered and encouraged to up skill and improve their competencies to enable them to deal with difficult tasks.

It was found that most of the respondents in this study were intrinsically motivated. Introjected and identified regulation, which are part of extrinsic motivation, were identified as important aspects in this study.

Feelings of competence, relatedness and autonomy encourage autonomous motivation by allowing leaders to act from the underlying self (Graves & Luciano 2013). Furthermore, leaders expect an environment of emotional support, warmth, friendliness and trust, which ensures a conducive working environment (Wipulanusat, Panuwatwanich & Stewart 2017). Leaders can achieve high levels of motivation when they feel related and can act effectively with personal initiative (Toode et al. 2014). Nurse leaders are expected to have substantial knowledge as well as leadership and management competencies in a changing environment (Kantanen et al. 2017). Intrinsic motivation is regulated by personal enjoyment, interest or pleasure, and it involves the performance of an activity for the inherent satisfaction of the activity (Naile & Salesho 2014). When leaders are intrinsically motivated, they tend to experience emotional well-being (Nunez & Leon 2016). Introjected regulation is somewhat less controlled and is represented by behaviours driven by internal rather than external rewards and punishment. The leader is motivated to avoid self-conscious emotions and obtain positive self-related affects and appraisals (Nie et al. 2015). Identified regulation is a more autonomous form of motivation in which the leader is motivated because the behaviour is congruent with the individual’s personal goals and values (Nie et al. 2015).

This study showed that nurse leaders engage in their tasks and activities because they are important to them. In fact, they find their work exciting and interesting. They also show pride in their job by achieving their goals.

Financial rewards and job security were not important motivators, and therefore it can be concluded that the respondents in this study were intrinsically motivated and that support, relatedness and competence were important motivators for them. Factors influencing the motivation of nurse leaders were determined, and therefore it can be concluded that the research problem was dealt with.

## Limitations

Because of the small sample size of 49 nurse leaders who participated in this study and the fact that the study was restricted to five hospitals in Gauteng in South Africa, the results cannot be generalised to other hospital groups. The male population of nurse leaders was not well represented.

## Recommendations

As identified from both literature and this study’s results, a few focus areas emerged, which must be addressed to ensure that nurse leaders stay motivated. The following recommendations can assist in the motivation of unit managers.

### Autonomy

It is allowing unit managers to work independently and make decisions on how to execute tasks (Miyata, Arai & Suga 2015), as well as supporting unit managers in decision-making skills and including them in strategic planning sessions (Papathanasiou et al. 2014).

### Relatedness

It is encouraging one-on-one relations-building sessions between unit managers and supervisors (Graves & Luciano 2013) and allowing unit managers to discuss their successes and challenges in group sessions. A sense of belonging will be established when team-building sessions with colleagues are encouraged (Utvær & Hagan 2016).

### Competence

It is establishing constructive feedback sessions on the unit managers’ competence on creating a culture of recognition for work well done (Van Dierendonck & Driehuizen 2015). Time for development must be allowed, and training needs should be identified. Mentoring by supervisors on expectations will assist in developing competence. Courses to up skill nurse leaders must be identified, addressed and budgeted for, and available conferences should also be budgeted for (Van Dierendonck & Driehuizen 2015).

### Support

It is showing interest in nurse leaders’ opinions and suggestions (Shariff 2015). By being involved in caring for their well-being (Wipulanusat et al. 2017), nurse leaders’ goals and values should be considered and included in their development plan (Solansky 2014). An open door policy with nurse leaders and supervisors must be encouraged (Wipulanusat et al. 2017). Confidence in their development should be shown by including them in succession planning (Van Dierendonck & Driehuizen 2015).

### Intrinsic motivation

It is ensuring that fun components are included in the nurse leaders’ work, by exploring what is exciting for these nurse leaders, and incorporating those at into their work (Chen & Bozeman 2013), as well as keeping their work interesting and having regular discussions with them to identify their specific needs (Naile & Salesho 2014).

### Identified and introjected regulation

It is encouraging nurse leaders to put effort into their jobs and also to align their work and personal values (Nie et al. 2015). Nurse leaders need to be allowed to prove that they can execute certain tasks successfully and should be appreciated for a task well done (Gagné et al. 2014). These achievements by nurse leaders must be recognised in public, and they should be encouraged to give feedback on positive outcomes (Witges & Scanlan 2014).

- Encouraging nurse leaders to put effort into their jobs and also to align their work and personal values.
- Nurse leaders need to be allowed to prove that they can execute certain tasks successfully and should be shown appreciation for a task well done.
- These achievements by nurse leaders must be recognised in public and they should be encouraged to give feedback on positive outcomes.

All of these activities will ensure that nurse leaders are motivated, and it will also keep them motivated.

## Conclusion

Nurse leaders who are not motivated cannot contribute to the profession, and therefore it is important to establish what motivators are important to leaders. Nursing is a constantly changing environment with new technology and policy changes being introduced all the time. Nurse leaders must therefore be empowered to adapt to these constant changes. This can happen only when nurse leaders are motivated and empowered. Motivation and overall work performance can be enhanced when attention is given to factors that are important to nurse leaders. Autonomous motivation is important for nurse leaders, and therefore it is important to allow them to make decisions and attempt to complete tasks in the manner they deem fit and to support their decision-making skills. When they are allowed to act autonomously, they can use their creativity, which can be advantageous for the organisation. This study determined that nurse leaders are intrinsically motivated and that introjected and identified regulation plays a role in their motivation. Autonomy, relatedness, competence and support are factors that influence their motivation. Recommendations that can enhance these factors and ensure that these nurse leaders stay motivated were listed.
